# Prediction and causal inference of hyperuricemia using gut microbiota

**DOI:** 10.1038/s41598-024-60427-6

**Published:** 2024-04-30

**Authors:** Yuna Miyajima, Shigehiro Karashima, Ren Mizoguchi, Masaki Kawakami, Kohei Ogura, Kazuhiro Ogai, Aoi Koshida, Yasuo Ikagawa, Yuta Ami, Qiunan Zhu, Hiromasa Tsujiguchi, Akinori Hara, Shin Kurihara, Hiroshi Arakawa, Hiroyuki Nakamura, Ikumi Tamai, Hidetaka Nambo, Shigefumi Okamoto

**Affiliations:** 1 Department of Clinical Laboratory Science, Faculty of Health Sciences, Institute of Medical, Pharmaceutical and Health Sciences, Kanazawa University, Kanazawa, Japan; 2 Institute of Liberal Arts and Science, Kanazawa University, Kakuma, Kanazawa, Ishikawa 920-1192 Japan; 3 Department of Health Promotion and Medicine of the Future, Kanazawa University, Kanazawa, Japan; 4 School of Electrical Information Communication Engineering, College of Science and Engineering, Kanazawa University, Kanazawa, Japan; 5 Institute for Frontier Science Initiative, Kanazawa University, Kanazawa, Japan; 6 Department of Bio-Engineering Nursing, Graduate School of Nursing, Ishikawa Prefectural Nursing University, Kahoku, Ishikawa Japan; 7 Faculty of Biology-Oriented Science and Technology, Kindai University, Kinokawa, Wakayama Japan; 8 Faculty of Pharmaceutical Sciences, Institute of Medical, Pharmaceutical and Health Sciences, Kanazawa University, Kanazawa, Japan; 9 Department of Hygiene and Public Health, Graduate School of Advanced Preventive Medical Sciences, Kanazawa University, Kanazawa, Japan; 10 School Introduction School of Entrepreneurial and Innovation Studies, College of Transdisciplinary Sciences for Innovation, Kanazawa University, Kanazawa, Japan; 11 Laboratory of Medical Microbiology and Microbiome, Department of Clinical Laboratory and Biomedical Sciences, Division of Health Sciences, Osaka University Graduate School of Medicine, 1-7 Yamadaoka, Suita, Osaka 565-0871 Japan

**Keywords:** Clinical microbiology, Epidemiology, Endocrine system and metabolic diseases

## Abstract

Hyperuricemia (HUA) is a symptom of high blood uric acid (UA) levels, which causes disorders such as gout and renal urinary calculus. Prolonged HUA is often associated with hypertension, atherosclerosis, diabetes mellitus, and chronic kidney disease. Studies have shown that gut microbiota (GM) affect these chronic diseases. This study aimed to determine the relationship between HUA and GM. The microbiome of 224 men and 254 women aged 40 years was analyzed through next-generation sequencing and machine learning. We obtained GM data through 16S rRNA-based sequencing of the fecal samples, finding that alpha-diversity by Shannon index was significantly low in the HUA group. Linear discriminant effect size analysis detected a high abundance of the genera *Collinsella* and *Faecalibacterium* in the HUA and non-HUA groups. Based on light gradient boosting machine learning, we propose that HUA can be predicted with high AUC using four clinical characteristics and the relative abundance of nine bacterial genera, including *Collinsella* and *Dorea*. In addition, analysis of causal relationships using a direct linear non-Gaussian acyclic model indicated a positive effect of the relative abundance of the genus *Collinsella* on blood UA levels. Our results suggest abundant *Collinsella* in the gut can increase blood UA levels.

## Introduction

Uric acid (UA) is present in the blood as the final metabolite of purine nucleic acid catabolism in humans, and its high concentration (> 7 mg/dL) is a risk factor for gout^1^. Hyperuricemia (HUA) is a risk factor for the development and progression of hypertension, atherosclerosis, insulin resistance, diabetes, and chronic kidney disease^2^, suggesting that the management of serum UA is clinically important. Gut microbiota (GM) varies widely among human populations but is closely associated with the development and progression of diabetes, obesity, atherosclerosis, and chronic kidney disease^3–6^. The importance of maintaining GM balance to regulate serum UA levels has been previously described^7^. Differences in the GM composition have also been reported in asymptomatic HUA^8^. GM data is complex, with potentially influential factors, such as geographic location, ethnicity, stress, age, and lifestyle^9^. Statistics and machine learning can explore and integrate disease-related features from complex data by identifying hidden patterns in correlations, and generating models that can accurately predict phenotypes^10^. Therefore, it has frequently been applied in GM research in recent years. Studies on GM and UA in humans are epidemiological studies and only examine associations, not causation. In other words, they do not assess the cause of the prediction, i.e. causality. One causal inference method that has been proposed to assess the causal structure of variables is the linear non-Gaussian acyclic model (LiNGAM)^11^. The aim of this study was to use LiNGAM to infer the causal relationship between GM and UA in Japanese adults.

## Results

### Clinical background

Two of the 488 participants who submitted fecal samples were excluded because they had less than 5000 sequences in the NGS analysis. Forty-one were taking UA-lowering drugs, antibiotics, steroids, bowel regulators, biocides, antibacterials, and proton pump inhibitors, and five were undergoing cancer treatment. Ten had missing health examination data, and 30 did not fast before blood collection. A total of 400 participants (176 men and 224 women) were included in the analysis.

### Differences in GM composition by HUA

**Figure 1:**
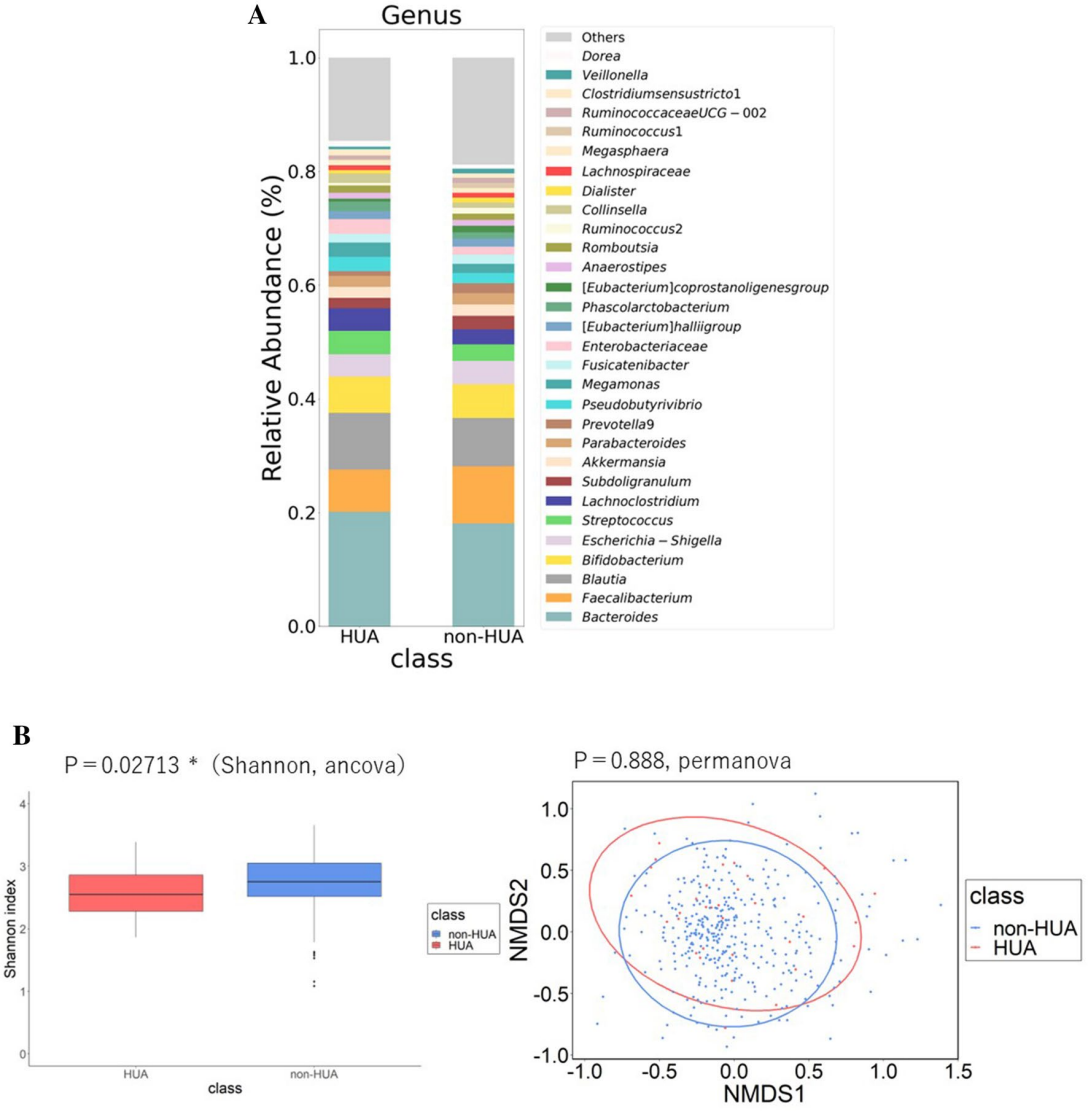
Differences in gut microbiota between HUA and non-HUA groups. (**A**) Comparison of relative abundance ratios at the phylum and genus level for the top 30 bacterial genera. (**B**) The difference in α-diversity calculated using the Shannon index (P = 0.027, Quade’s nonparametric ANCOVA). (**C**) Plot of β-diversity analysis calculated by NMDS ordering based on Bray–Curtis distance matrix. Red: HUA, blue: non-HUA. Ellipses represent 95% confidence intervals for each genus used in the analysis. (P = 0.888, PERMANOVA). *NMDS* non-metric multidimensional scaling, *ANCOVA* analysis of covariance, *HUA* hyperuricemia, *PERMANOVA* permutation multivariate analysis of variance.

Table 1 shows the clinical characteristics of the HUA (UA > 7.0 mg/dL in the blood) and non-HUA groups. There were significant differences in BMI, waist circumference, UA, S-Cre, eGFR, and frequency of alcohol consumption between the two groups. The composition of the top 30 genera of intestinal bacteria in the two groups at the level of genus is shown in Fig. 1A. The Shannon index was significantly reduced in the HUA group (Fig. 1B, P = 0.027, ANCOVA), and non-metric multidimensional scaling analysis using the Bray–Curtis distance (diversity) showed no significant difference in gut bacterial composition between the two groups (Fig. 1C).

**Table 1:** Characteristics of study participants.

Characteristic	Hyperuricemia	Non-hyperuricemia	*P*-value
n	32	368	
Age, (years)	63.5 (56.5 ± 68.0)	64 (56.0 ± 70.0)	0.530
Sex, (female %)	9.4	60.1	< 0.001
BMI, (kg/m^2^)	25.3 (23.28 ± 26.7)	23.0 (20.7 ± 24.9)	< 0.001
Waist circumference (cm)	88.55 (84.25 ± 94.6)	83.10 (76.70 ± 88.80)	< 0.001
Uric acid (mg/dL)	7.6 (7.28 ± 8.33)	4.85 (4.10 ± 5.70)	< 0.001
S-Cre (mg/dL)	1.00 (0.89 ± 1.08)	0.74 (0.65 ± 0.86)	< 0.001
eGFR (mL/min/1.73 m^2^)	59.09 (53.63 ± 66.38)	68.19 (61.62 ± 75.58)	< 0.001
Frequency of alcohol consumption (day/week)	6.00 (0.75 ± 7.00)	0.0 (0.0 ± 0.0)	< 0.001
Current smoking (n/day)	0.0 (0.0 ± 0.0)	0.0 (0.0 ± 0.0)	0.175
DM (%)	18.8	9.5	0.099
DL (%)	15.6	23.3	0.317
HT (%)	34.4	31.3	0.716
CVD (%)	3.1	3.3	0.967
Stroke (%)	0	1.6	0.468

The P-values were calculated by covariance analysis (ANCOVA or Quade’s non-parametric ANCOVA). *ANCOVA* analysis by covariance, *BMI* body mass index, *S-Cre* Serum creatinine, *eGFR* estimated glomerular filtration rate, *DM* diabetes mellitus, *DL* dyslipidemia, *HT* hypertension, *CVD* Cardiovascular Disease.

### GM associated with HUA

**Figure 2:**
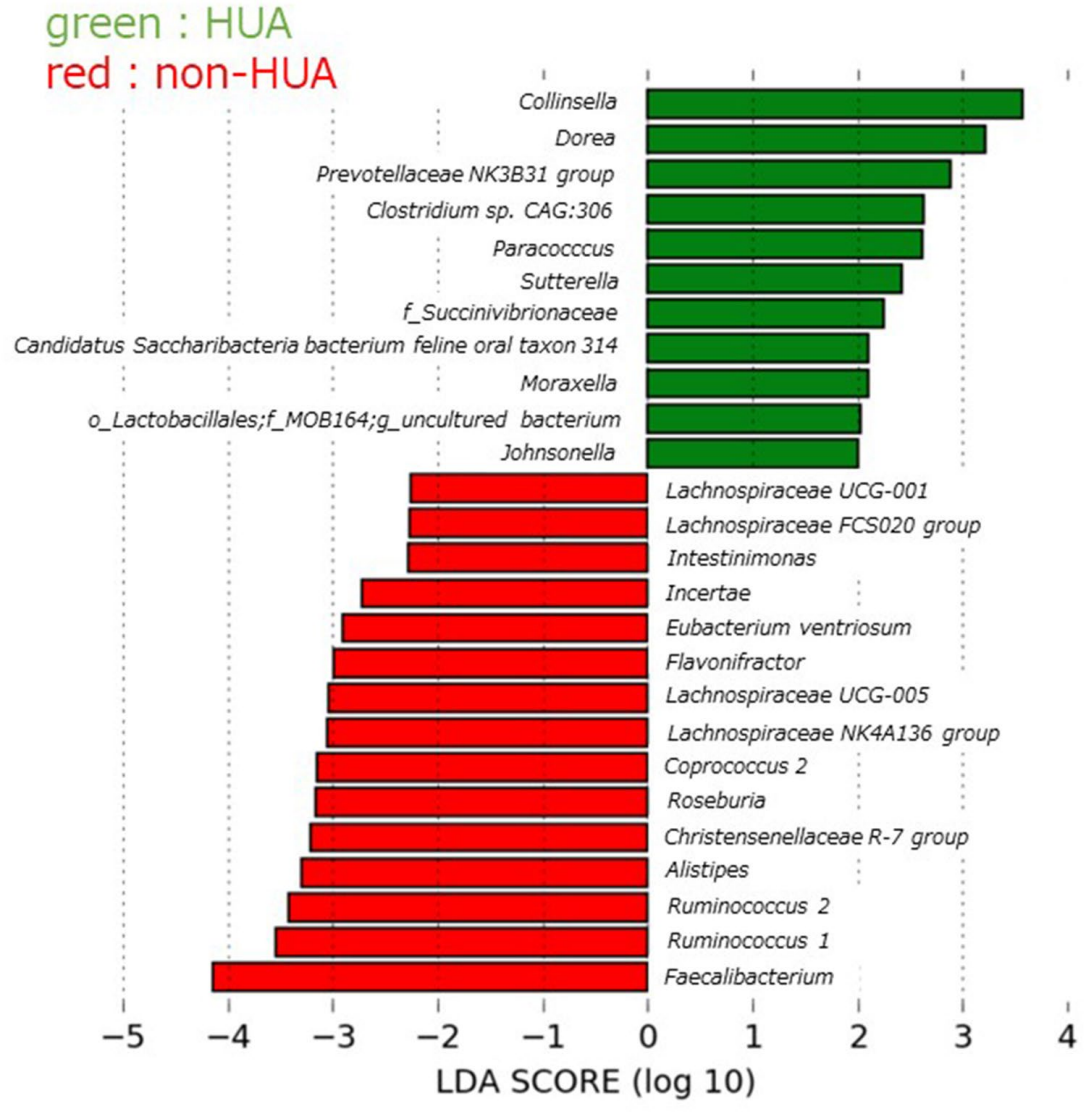
Identification of the intestinal bacteria involved in HUA. LEfSe analysis of the top 436 bacterial species, with LDA score = 2.0 as the cutoff value. *HUA* hyperuricemia, *non-HUA* non-hyperuricemia, *LEfSe* linear discriminant analysis effect size.

LEfSe analysis of all 436 bacteria (Fig. 2) showed that 11 and 15 that were significantly high in the HUA and non-HUA groups. The bacteria with the highest linear discriminant analysis (LDA) score in the HUA group was the genus *Collinsella* (LDA score = 3.569, P = 0.013), and the bacteria with the highest LDA values in the non-HUA group were *Faecalibacterium* (LDA score = 4.138, P = 0.033).

### Prediction of HUA patients by intestinal bacteria

**Figure 3:**
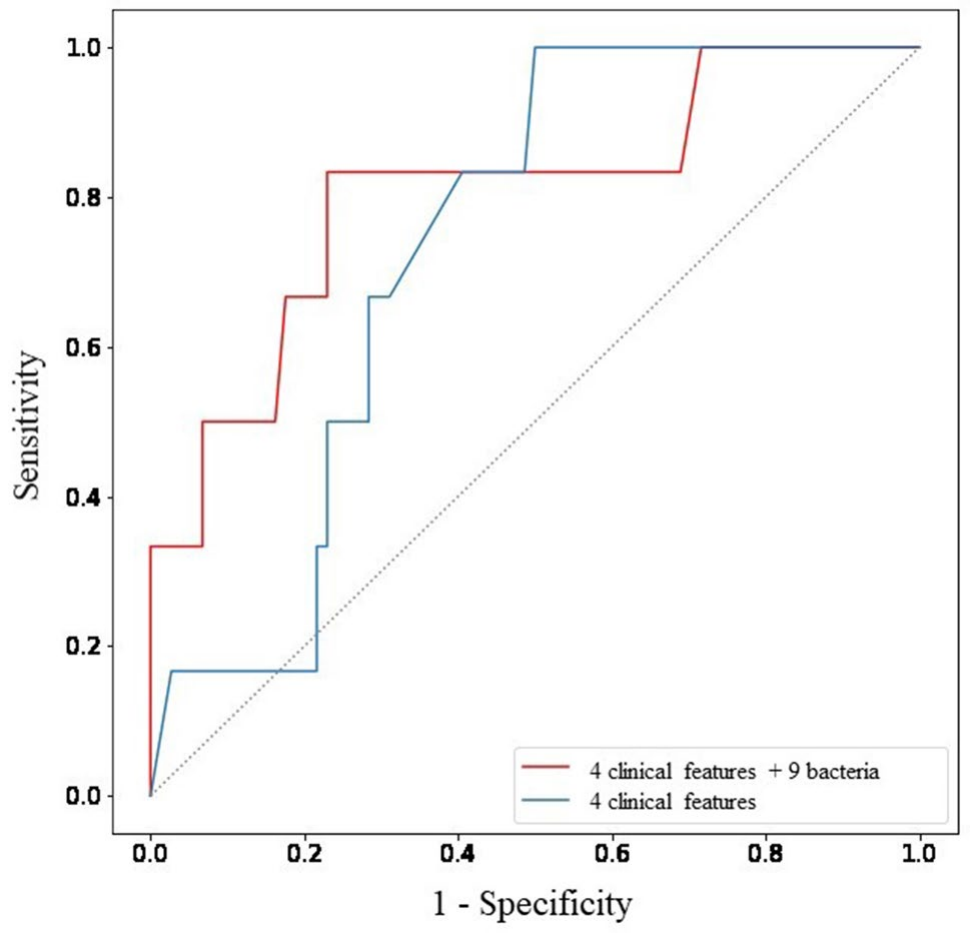
Receiver operating characteristic (ROC) curve curves for models predicting the presence or absence of HUA. The performance of the model using 13 characteristics, including bacterial genera, is shown in red. The performance of the model using only four variables, age, BMI, waist circumference and frequency of alcohol consumption, is shown in blue. The ROC curve of the model with the median AUC out of 50 cross-validations is shown.

LGBM was used to select the GM that contributed to the prediction of HUA and non-HUA classifications. Feature selection was used to construct the most accurate model from 37 features, including 11 basic clinical traits (Age, Sex, BMI, Waist, Frequency of alcohol consumption, Frequency of smoking, medical history (Diabetes mellitus, Hypertension, dyslipidemia, Cardio Vascular Disease, Stroke)) and 26 bacterial species (*Faecalibacterium*, *Ruminococcus 2*, *Collinsella*, *Ruminococcus 1*, *Dorea*, *Alistipes*, *Roseburia*, *Incertae Sedis*, *Lachnospiraceae NK4A136 group*, *Christensenellaceae R-7 group*, *Lachnospiraceae UCG-005*, *Coprococcus 2*, *Eubacterium ventriosum group*, *Flavonifractor*, *Prevotellaceae NK3B31 group*, *Sutterella*, *Lachnospiraceae FCS020 group*, *Intestinimonas*, *Lachnospiraceae UCG-001*, *Stenotrophomonas*, and *Paracocccus*, *Clostridium *sp.* CAG:306*). Feature selection was performed to build the most accurate model. As a result, a highly accurate HUA prediction model with AUC of 0.829 ± 0.043 (Fig. 3), ACC of 0.725 ± 0.080, sensitivity of 0.757 ± 0.148, specificity of 0.723 ± 0.100, and PPV of 0.201 ± 0.038 was constructed with 13 features (four clinical traits and nine bacteria) that contribute significantly to HUA prediction, as shown in Table 2. In contrast, the performance of the prediction model using only four clinical features was AUC 0.740 ± 0.032, ACC 0.630 ± 0.070, sensitivity 0.780 ± 0.075, specificity 0.617 ± 0.078 and PPV 0.157 ± 0.039.

**Table 2:** List of features used in the HUA prediction algorithm showing the highest AUC in LGBM.

Clinical information	bacteria
Age	*Ruminococcus 2*
BMI	*Collinsella*
Waist circumference	*Dorea*
Frequency of alcohol consumption	*Alistipes*
	*Roseburia*
	*Lachnospiraceae NK4A136 group*
	*Flavonifractor*
	*Sutterella*
	*Lachnospiraceae FCS020 group*

*BMI* body mass index.

### Correlation between UA levels and GM

**Figure 4:**
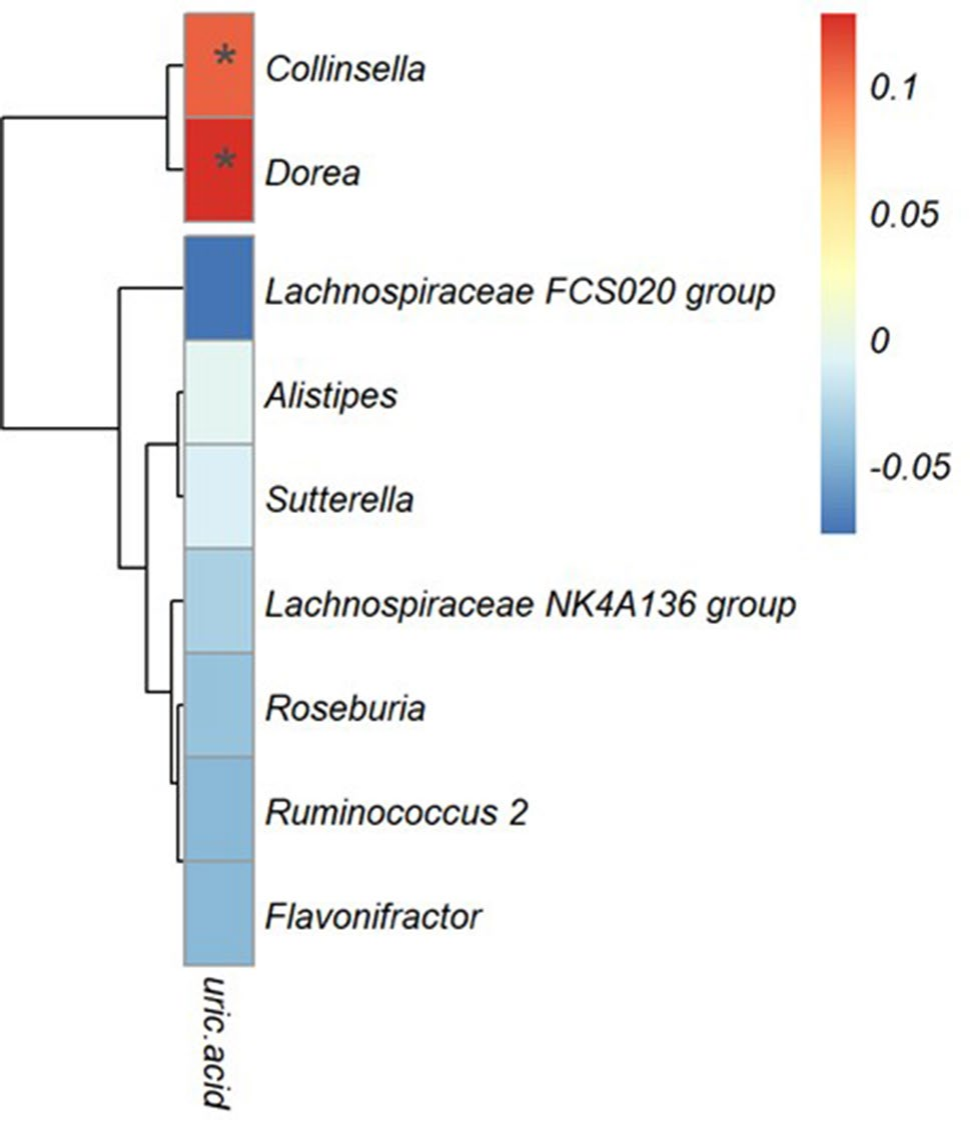
Correlation between serum UA levels and bacterial genus abundance ratios. The bacteria shown in the figure are the nine bacterial genera that could be predicted to have the highest AUC in LGBM. Spearman's correlation coefficient value determines the color intensity of the heatmap. Red: positive correlation, blue: negative correlation. (*P < 0.05). The correlation matrix was visualized as a heatmap using "pheatmap in R.

Figure 4 shows the correlation between serum UA levels and the relative abundance of the nine intestinal bacteria selected in the LGBM; as the heatmap shows, a significant correlation was demonstrated between the genera *Collinsella* and *Dorea* and serum UA levels (Fig. 4). No significant correlation was observed between these two intestinal genera and renal function indices in serum, such as eGFR and S-Cre, other than UA (Supplementary Fig. 1).

### Causal relationship between serum UA levels and Intestinal bacteria using Direct LiNGAM

**Figure 5:**
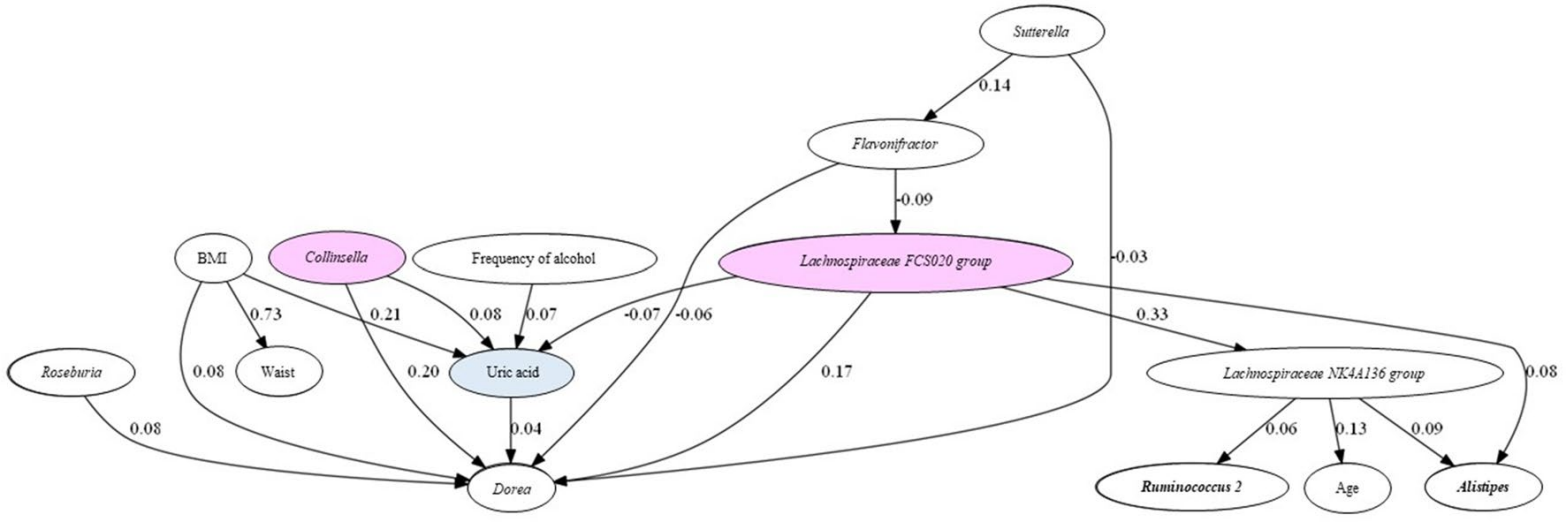
Causal inference between serum UA levels and GM by LinGAM. Arrows indicate the direction of causality between the two indices. Values are standardized partial regression coefficients. Red: bacteria with an inferred causal relationship with UA; blue: serum UA level. Numerical values are absolute values of the partial regression coefficients.

LiNGAM algorithm was used to infer the causal relationship between serum UA levels and intestinal bacterial abundance ratios. The inferred causal diagrams, causal ranks, and partial regression coefficients for the serum UA levels of the nine intestinal bacteria selected in LGBM are shown in Fig. 5. The arrows represent the inferred causal relationships of two linkage indices with non-zero partial regression coefficients: the genus *Collinsella* had a positive effect on UA levels (coefficient = 0.08), while the *Lachnospiraceae FCS020 group* had a negative effect (coefficient = − 0.07). The results showed that the genus *Dorea*, which positively correlated with serum UA levels, had a possible positive effect from the genus *Collinsella* (coefficient = 0.20). The inferred causality relationship, including other serum renal function indices (eGFR and S-Cre), also showed that the genus *Collinsella* positively influenced serum UA levels (coefficient = 0.06) (Supplementary Fig. 2).

## Discussion

Statistical analysis and machine learning revealed associations between specific gut bacteria and HUA and inferred a causal relationship. The genera *Collinsella*, *Dorea,* and *Lachnospiraceae* FCS020 group were identified as characteristic bacteria involved in HUA. Direct LiNGAM suggested that the genera *Collinsella* and *Lachnospiraceae* FCS020 group may alter serum UA levels. The presence or absence of HUA can be accurately predicted using general laboratory information and gut bacteria data.

Particularly, the genus *Collinsella* is presumed to have a direct causal relationship with UA. *Collinsella aerofaciens*, a representative species of the genus *Collinsella*, is abundant in the intestinal flora of Asians^12^ and produces butyric, formic, lactic (LA), and acetic acids^13^. Indeed, *C. aerofaciens* has been reported to affect host health and disease^14^, and there are currently no reports of *Collinsella* spp. increasing or decreasing in subjects with HUA or affecting serum UA levels to become a risk factor for HUA.

There are four possible mechanisms by which *Collinsella* spp. modulate host serum UA levels. First, *Collinsella* spp. directly produce UA. Second, *Collinsella* spp. indirectly inhibit UA degradation by other bacteria. Finally, the metabolites produced by *Collinsella* spp. reduce renal and intestinal excretion of UA.

*Collinsella* spp. harbor gene sequences for hypoxanthine, the precursor of UA, and xanthine dehydrogenase, which converts xanthine into UA (NCBI database).

A known bacterial catabolic pathway for UA is the allantoin pathway, which involves the interconversion of 5-hydroxyisouric acid, 2-oxo-4-hydroxy-4-carboxy-4-carboxy-5-ureidoimidazoline, and allantoin in three steps and is readily degraded to ammonia^15^. Many bacterial species utilize this metabolic pathway. *Lactobacillus brevis* (DM9218) and *Lactobacillus gasseri* (PA-3) have also potential as probiotics to improve HUA by degrading intermediates of purine metabolism^16^. *Lactobacillus gasseri* (PA-3) is a bacterium recently found in yogurt and other products, suggesting that dietary habits may be affecting UA levels via GM^17^. If *Corinella* spp. can inhibit the activity and growth of enterobacteria that cause the degradation of interstitial UA, this may constitute a mechanism to increase UA levels in the host.

Serum UA is excreted from the kidneys and intestinal tract. Thus, indole and LA from *Corinella* spp. may cause additional renal UA excretion. Indole and LA have been found to inhibit serum UA excretion when the blood UA levels increase^18–20^. *Corinella* spp. possess tryptophanase, which metabolizes tryptophan to indole. The indole produced is transferred to the liver, where it is converted to indoxyl sulfate and is thought to be responsible for the aggravation of renal and vascular diseases^18,19^. Kurihara et al. reported that *C. aerofaciens* produces sufficient LA^20^. However, bacterial species, such as Enterococcus faecalis and Bacteroides intestinalis, have been reported to produce particularly high lactate levels, suggesting that *Collinsella* spp. may not be the only cause of HUA via this mechanism.

Loss of function of the ATP-binding cassette transporter G2 (ABCG2), which is abundantly expressed in the intestinal tract, mainly in the ileum, has been reported to cause HUA and gout^21,22^; ABCG2 excretes not only UA but also the aforementioned indole sulfate^23,24^. The mechanisms by which *Chorinella* spp. regulate host UA levels require further study.

In addition to *Collinsella* spp., *Lachnospiraceae* FCS020 group also showed the potential to reduce serum UA levels; *Lachnospiraceae* FCS020 group was significantly reduced in the HUA group, similar to *Lachnospiraceae bacteriaceae*^25^. Bacterial species in this family may act as protective factors against HUA.

In general, seafood, soy products, and beer, when consumed in excess, tend to increase uric acid levels^26^. In addition, consumption of probiotic-containing beverages such as yogurt and Yakult may affect the intestinal bacteria associated with HUA. Therefore, using information about participants daily eating habits may improve the accuracy and reliability of models predicting HUA.

This study has certain limitations. First, the sample size was small. Overall, 400 samples were included in the analysis, of which 31 were from patients with HUA. In addition to the small sample size, the lack of data in another population did not allow us to conduct an external validation to assess the performance of the forecasting model by LGBM. Second, analysis was performed at the bacterial genus level in the 16s rRNA V3-V4 region. A more detailed classification at the bacterial species level, rather than at the bacterial genus level, would reveal changes in serum UA levels, which would be more beneficial for clinical applications such as dietary and other probiotic interventions. Finally, although this study was predictive and inferential based on observational data and took confounding factors into account to the greatest extent possible, the influence of potential confounding factors cannot be completely ruled out.

In conclusion, we confirmed that the genus *Collinsella* may be the GM most causally related to serum UA levels in the present population. This suggests that maintaining a low ratio of certain gut bacteria may lead to the maintenance of serum UA levels, reducing the risk of HUA. In the future, it may be possible to identify GM compositions that improve UA metabolism and contribute to the prevention of HUA. The discovery of prebiotics that affect *Chorinella* spp. and increasing the number of gut bacteria that antagonise *Chorinella* spp. could be a new therapeutic strategy for patients with HUA. Further studies are required to elucidate the detailed mechanisms of action of the GM in HUA.

## Materials and methods

### Participants

The participants were 488 residents (224 men and 254 women) aged 40 years or older, of Shika-machi, Hakui-gun, Ishikawa Prefecture, Japan, whose fecal samples were collected during a health checkup in January 2018 and 2020 (n = 254: 115 men, 131 women, 8 unknown) and January 2020 (n = 234: 109 men, 123 women, 2 unknown). The patients were divided into two groups, HUA, and non-HUA groups, based on a criterion^1^: the HUA group with serum UA > 7.0 mg/dL in blood. We excluded following patients (1) who had been taking UA-lowering drugs, antibiotics, steroids, bowel regulators, biocides, antibacterial agents, and proton pump inhibitors; (2) who had been undergoing any treatment for cancer, (3) who had eaten within 10 h at the time of blood collection; and (4) whose diagnostic data were missing.

### Data source

Data from the Shika-machi Super Preventive Health Examination, a population survey aimed at establishing preventive methods for lifestyle-related diseases, were used. The survey was conducted twice, in January 2018 and January 2020. The four model districts selected from the Shika area were Horimatsu, Higashimasuho, Tsuchida, and Higashiki^27,28^.

### Ethical considerations

This study was approved by the Kanazawa University Hospital Human Research Ethics Committee (approval number: 1491) and conducted in accordance with the principles of the Declaration of Helsinki and the Kanazawa University Microbial Safety Management Regulations. After providing an overview of the study to all participants at the time of physical examination, written informed consent prior to GM collection was obtained. The fecal samples were processed in a non-proliferation level 2 (P2) laboratory.

### Data collection

The Super-Preventive Health Checkup data (Shika Town) regarding parameters such as age, sex, medical history, medication status, and alcohol consumption/smoking status were collected using a questionnaire. The body mass index (BMI) was calculated by dividing the current weight (kg) by the square of the height (m^2^). After fasting for 12 h, venous blood was collected and serum UA levels (s-UA), and serum creatinine (S-Cre) were measured. Estimated glomerular filtration rate (eGFR) was calculated with S-Cre as in previous articles^28^.

### Fecal sample collection and DNA extraction

Fecal samples were collected from 488 participants using the method described previously^29^. The stool surface samples were collected independently by the participants using clean paper (AZ-ONE, Osaka, Japan) and a clean spatula with a plastic tube (AZ-ONE, Japan). The collected fecal samples were kept on ice and transported to the laboratory. The samples were stored at − 80 °C until DNA extraction. The total DNA extraction was performed using the NucleoSpin® DNA Stool (Machery Nagel, Dürren, Germany).

### Next-generation sequencing

The DNA extracted from the GM was processed for identification of the 16S rRNA gene sequence by NGS, using a previously described method^28^. The 16S rRNA gene was amplified using the 1st PCR primers (F: 5′-TCG TCG GCA GCG TCA GAT GTG TAT AAG AGA CAG CCT ACG GGN GGC WGC AG-3′; R: 5′-GTC TCG TGG GCT CGG AGA TGT GTA TAA GAG ACA GGA CTA CHV GGG TAT CTA ATC C-3′)^11^ (Hokkaido system science Co., Ltd., Osaka, Japan). Ex Taq® hot-start version (TaKaRa Bio Inc., Shiga, Japan) and TaKaRa PCR Thermal Cycler Dice® Gradient (TaKaRa Bio Inc., Shiga, Japan) were used to amplify the V3-V4 region of the 16S rRNA gene. Polymerase chain reaction (PCR) products were purified using Agencourt AMPure XP magnetic beads (Beckman Coulter, Inc., CA, USA). The concentrations of the resultant PCR products were measured using the Qubit®dsDNA HS Assay Kit and Qubit® 3.0 Fluorometer (Thermo Fisher Scientific). All the purified PCR products were indexed and sequenced using MiSeq (Illumina, Inc., CA, USA) with MiSeq Reagent Kit version 3 and PhiX Control v3 (Illumina).

### Microbiome analysis

For microbiome analysis, QIIME2 software was used^30^. Demultiplexed paired-end sequence data were denoised with DADA2, and the Silva 16S rRNA database (release 132)^31^ naïve Bayes classifier was used for ASV classification. Samples with fewer than 5000 sequences were removed from the analysis.

### Statistical analysis

Python (version 3.8.8) with the scikit-learn package (version 0.24.1)^32^ or R, using R-studio (version 4.1.1) (Rstudio, Boston, MA, United States), was used for statistical analysis and machine learning.

The clinical information of the participants was tested for normality of distribution using the Shapiro–Wilk test. Normally distributed data are presented as mean ± standard deviation, and non-normally distributed data are presented as median (25th–75th percentile). The differences in the clinical information between the groups were tested for significance using one-way analysis of covariance (ANCOVA) for normally distributed data and ANCOVA with rank ordering (Quade’s non-parametric ANCOVA)^33^ for non-normally distributed data. The significance level of all the tests was set at *P* < 0.05. Alpha diversity, the beta diversity and similarity between each participant group was assessed using the non-metric multidimensional scaling analysis with the Bray–Curtis of R’s “package vegan” and the permutation multivariate analysis of variance^34^. A linear discriminant analysis effect size (LEfSe) was used to identify the GMs associated with HUA^35^. Light Gradient Boosting Machine (LGBM)^36^, a model was built to predict HUA. The model was cross-validated 5 times using “Stratified K-Fold” to split the training/test set data. Ensemble learning was performed 100 times using “bagging”. The five measures of accuracy including area under the receiver operating characteristic curve (AUC), accuracy (ACC), sensitivity, specificity, and positive predictive value (PPV) were averaged over 10 model building cycles. The features used for model building were 11 basic clinical information and 26 bacterial genera shown to be significantly associated with HUA in the LEfSe analysis. “Feature selection” was also performed to build a model with the highest AUC. Correlation coefficients and P-values were calculated using Spearman’s rank correlation coefficient in R’s “Package ppcor” after adjusting for the variables listed above. The correlation coefficients were plotted using “Package pheatmap” in R. The Direct LiNGAM was built using “LiNGAM” in Python^11,37^. The bacterial genus features used for the Direct LiNGAM were those used for feature selection in the LGBM model, the model showing the highest AUC. The partial regression coefficients shown were normalized to a minimum value of 0 and a maximum value of 1 using “MinMaxScaler” in the scikit-learn package.

### Ethics statement

This study was reviewed and approved by the Ethics Committee for Human Studies at Kanazawa University Hospital (approval number: 1491). The participants provided written informed consent.

### Supplementary Information

Supplementary Legends.

Supplementary Table S1.

Supplementary Figure S1.

Supplementary Figure S2.

## Data Availability

The raw data of the sequencing was registered at DNA Data Bank of Japan (DDBJ) (Number DRA016467). Supplementary Table 1 listed the patient IDs analyzed in the study.
