# Supplementary material for: Prediction and causal inference of hyperuricemia using gut microbiota
Source: Sci Rep. 2024 Apr 30;14:9901. doi: 10.1038/s41598-024-60427-6 (PMC11061287; doi:10.1038/s41598-024-60427-6)
Supplement: Supplementary file 1 — Supplementary Legends. [file 41598_2024_60427_MOESM1_ESM.docx]

# Supplementary Figure legends

**Supplementary Figure 1**. Correlation between UA, eGFR, and S-Cre, and the ratio of GM presence. Spearman's correlation coefficient determines the color intensity of the heatmap. Red: positive correlation, blue: negative correlation. (*: P < 0.05).

**Supplementary Figure 2**. Causal inference between uric acid levels and GM, including S-Cre and eGFR. Arrows indicate the causal relationship between two connected indicators. Values are standardized partial regression coefficients. Red: bacteria with an estimated causal relationship with UA; blue: serum UA level. Values are absolute values of partial regression coefficients
