# Supplementary material for: Prediction and causal inference of hyperuricemia using gut microbiota
Source: Sci Rep. 2024 Apr 30;14:9901. doi: 10.1038/s41598-024-60427-6 (PMC11061287; doi:10.1038/s41598-024-60427-6)
Supplement: Supplementary file 2 — Supplementary Table S1. [file 41598_2024_60427_MOESM2_ESM.docx]

Supplementary Material

**Prediction and causal inference of hyperuricemia using gut microbiota**

Yuna Miyajima^1†^, Shigehiro Karashima^2*^, Ren Mizoguchi^3†^, Masaki Kawakami^4^, Kohei Ogura^5^, Kazuhiro Ogai^6^, Aoi Koshida^5^, Yasuo Ikagawa^5^, Yuta Ami^7^, Qiunan Zhu^8^, Hiromasa Tsujiguchi^9^, Akinori Hara^9^, Shin Kurihara^7^, Hiroshi Arakawa^8^, Hiroyuki Nakamura^9^, Ikumi Tamai^8^, Hidetaka Nambo^10^, Shigefumi Okamoto^11*^

^1^ Department of Clinical Laboratory Science, Faculty of Health Sciences, Institute of Medical, Pharmaceutical and Health Sciences, Kanazawa University, Kanazawa, Japan

^2^ Institute of Liberal Arts and Science, Kanazawa University, Kanazawa, Japan

^3^ Department of Health Promotion and Medicine of the Future, Kanazawa University, Kanazawa, Japan

^4^ [School of Electrical Information Communication Engineering](http://www.ec.t.kanazawa-u.ac.jp/index.en.html), [College of Science and Engineering](http://www.kanazawa-u.ac.jp/e/academics/college/cse/), [Kanazawa University](http://www.kanazawa-u.ac.jp/e/), Kanazawa, Japan.

^5^ Institute for Frontier Science Initiative, Kanazawa University, Kanazawa, Japan

^6^ Department of Bio-engineering Nursing, Graduate School of Nursing, Ishikawa Prefectural Nursing University, Kahoku, Ishikawa, Japan

^7^ Faculty of Biology-Oriented Science and Technology, Kindai University, Kinokawa, Wakayama, Japan.

^8^ Faculty of Pharmaceutical Sciences, Institute of Medical, Pharmaceutical and Health Sciences, Kanazawa University, Kanazawa, Japan

^9^ Department of Hygiene and Public Health, Graduate School of Advanced Preventive Medical Sciences, Kanazawa University, Kanazawa, Japan

^10^ School introduction School of Entrepreneurial and Innovation Studies, College of Transdisciplinary Sciences for Innovation, Kanazawa University, Kanazawa, Japan.

^11^ Laboratory of Medical Microbiology and Microbiome, Department of Clinical Laboratory and Biomedical Sciences, Division of Health Sciences, Osaka University Graduate School of Medicine

*** Correspondence:**

Shigehiro Karashima, MD, PhD

Institute of Liberal Arts and Science, Kanazawa University, Kanazawa, Japan

Kakuma, Kanazawa, Ishikawa, 920-1192, Japan

Phone: +81 76-264-5802, FAX: +81-76-234-4271

E-mail: skarashima@staff.kanazawa-u.ac.jp

Shigefumi Okamoto, DDS, PhD

Laboratory of Medical Microbiology and Microbiome, Department of Clinical Laboratory and Biomedical Sciences, Division of Health Sciences, Osaka University Graduate School of Medicine,
1-7 Yamadaoka, Suita, Osaka 565-0871, Japan.
Phone/Fax: +81 6 6879 2612,

E-mail: [sokamoto@sahs.med.osaka-u.ac.jp](mailto:sokamoto@sahs.med.osaka-u.ac.jp)

# Supplementary Figures and Tables

## Supplementary Figures

**Supplementary Figure 1**. Correlation between UA, eGFR, and S-Cre, and the ratio of GM presence. Spearman's correlation coefficient determines the color intensity of the heatmap. Red: positive correlation, blue: negative correlation. (*: P < 0.05).

**Supplementary Figure 2**. Causal inference between uric acid levels and GM, including S-Cre and eGFR. Arrows indicate the causal relationship between two connected indicators. Values are standardized partial regression coefficients. Red: bacteria with an estimated causal relationship with UA; blue: serum UA level. Values are absolute values of partial regression coefficients.

## Supplementary Table

**Supplementary Table 1.** List of Bio sample IDs used for the analysis from the raw sequencing registered in the DNA Data Bank of Japan (DDBJ) (Numbers DRA016467). Supplementary Table 1 listed the patient IDs analyzed in the study.

| HUA | non-HUA | | | |
| --- | --- | --- | --- | --- |
| SAMD00620868 | SAMD00620859 | SAMD00620990 | SAMD00621117 | SAMD00621259 |
| SAMD00620907 | SAMD00620860 | SAMD00620991 | SAMD00621118 | SAMD00621260 |
| SAMD00620978 | SAMD00620862 | SAMD00620992 | SAMD00621119 | SAMD00621261 |
| SAMD00620988 | SAMD00620863 | SAMD00620994 | SAMD00621120 | SAMD00621263 |
| SAMD00620998 | SAMD00620864 | SAMD00620997 | SAMD00621121 | SAMD00621264 |
| SAMD00621049 | SAMD00620866 | SAMD00620999 | SAMD00621123 | SAMD00621265 |
| SAMD00621085 | SAMD00620867 | SAMD00621000 | SAMD00621125 | SAMD00621267 |
| SAMD00621107 | SAMD00620870 | SAMD00621003 | SAMD00621127 | SAMD00621268 |
| SAMD00621109 | SAMD00620871 | SAMD00621004 | SAMD00621128 | SAMD00621269 |
| SAMD00621112 | SAMD00620872 | SAMD00621005 | SAMD00621129 | SAMD00621270 |
| SAMD00621131 | SAMD00620873 | SAMD00621006 | SAMD00621130 | SAMD00621272 |
| SAMD00621156 | SAMD00620874 | SAMD00621007 | SAMD00621133 | SAMD00621274 |
| SAMD00621164 | SAMD00620875 | SAMD00621008 | SAMD00621134 | SAMD00621275 |
| SAMD00621176 | SAMD00620877 | SAMD00621009 | SAMD00621135 | SAMD00621276 |
| SAMD00621180 | SAMD00620878 | SAMD00621010 | SAMD00621136 | SAMD00621279 |
| SAMD00621185 | SAMD00620879 | SAMD00621012 | SAMD00621137 | SAMD00621280 |
| SAMD00621189 | SAMD00620880 | SAMD00621013 | SAMD00621139 | SAMD00621281 |
| SAMD00621193 | SAMD00620881 | SAMD00621014 | SAMD00621140 | SAMD00621282 |
| SAMD00621194 | SAMD00620884 | SAMD00621015 | SAMD00621141 | SAMD00621283 |
| SAMD00621214 | SAMD00620885 | SAMD00621016 | SAMD00621142 | SAMD00621284 |
| SAMD00621231 | SAMD00620886 | SAMD00621017 | SAMD00621143 | SAMD00621285 |
| SAMD00621240 | SAMD00620887 | SAMD00621018 | SAMD00621144 | SAMD00621286 |
| SAMD00621253 | SAMD00620888 | SAMD00621019 | SAMD00621145 | SAMD00621287 |
| SAMD00621258 | SAMD00620889 | SAMD00621020 | SAMD00621146 | SAMD00621288 |
| SAMD00621262 | SAMD00620890 | SAMD00621021 | SAMD00621148 | SAMD00621289 |
| SAMD00621271 | SAMD00620892 | SAMD00621022 | SAMD00621150 | SAMD00621290 |
| SAMD00621277 | SAMD00620893 | SAMD00621023 | SAMD00621151 | SAMD00621291 |
| SAMD00621297 | SAMD00620894 | SAMD00621024 | SAMD00621154 | SAMD00621293 |
| SAMD00621318 | SAMD00620895 | SAMD00621025 | SAMD00621155 | SAMD00621294 |
| SAMD00621322 | SAMD00620896 | SAMD00621026 | SAMD00621157 | SAMD00621295 |
| SAMD00621324 | SAMD00620898 | SAMD00621027 | SAMD00621158 | SAMD00621296 |
| SAMD00621327 | SAMD00620899 | SAMD00621029 | SAMD00621159 | SAMD00621298 |
|  | SAMD00620900 | SAMD00621030 | SAMD00621160 | SAMD00621299 |
|  | SAMD00620901 | SAMD00621032 | SAMD00621161 | SAMD00621300 |
|  | SAMD00620902 | SAMD00621034 | SAMD00621162 | SAMD00621301 |
|  | SAMD00620903 | SAMD00621035 | SAMD00621163 | SAMD00621302 |
|  | SAMD00620904 | SAMD00621036 | SAMD00621165 | SAMD00621303 |
|  | SAMD00620905 | SAMD00621037 | SAMD00621166 | SAMD00621306 |
|  | SAMD00620908 | SAMD00621038 | SAMD00621167 | SAMD00621307 |
|  | SAMD00620909 | SAMD00621039 | SAMD00621168 | SAMD00621308 |
|  | SAMD00620910 | SAMD00621040 | SAMD00621169 | SAMD00621309 |
|  | SAMD00620911 | SAMD00621041 | SAMD00621170 | SAMD00621310 |
|  | SAMD00620912 | SAMD00621044 | SAMD00621171 | SAMD00621311 |
|  | SAMD00620913 | SAMD00621045 | SAMD00621173 | SAMD00621312 |
|  | SAMD00620914 | SAMD00621046 | SAMD00621177 | SAMD00621313 |
|  | SAMD00620915 | SAMD00621047 | SAMD00621178 | SAMD00621314 |
|  | SAMD00620916 | SAMD00621050 | SAMD00621181 | SAMD00621315 |
|  | SAMD00620917 | SAMD00621051 | SAMD00621182 | SAMD00621319 |
|  | SAMD00620918 | SAMD00621052 | SAMD00621183 | SAMD00621320 |
|  | SAMD00620919 | SAMD00621053 | SAMD00621184 | SAMD00621321 |
|  | SAMD00620922 | SAMD00621054 | SAMD00621188 | SAMD00621323 |
|  | SAMD00620923 | SAMD00621055 | SAMD00621190 | SAMD00621325 |
|  | SAMD00620924 | SAMD00621056 | SAMD00621191 | SAMD00621326 |
|  | SAMD00620925 | SAMD00621058 | SAMD00621192 | SAMD00621328 |
|  | SAMD00620926 | SAMD00621059 | SAMD00621195 | SAMD00621329 |
|  | SAMD00620927 | SAMD00621060 | SAMD00621196 | SAMD00621330 |
|  | SAMD00620928 | SAMD00621061 | SAMD00621197 | SAMD00621332 |
|  | SAMD00620930 | SAMD00621062 | SAMD00621199 | SAMD00621334 |
|  | SAMD00620933 | SAMD00621063 | SAMD00621200 | SAMD00621336 |
|  | SAMD00620934 | SAMD00621064 | SAMD00621201 | SAMD00621337 |
|  | SAMD00620935 | SAMD00621065 | SAMD00621202 | SAMD00621339 |
|  | SAMD00620936 | SAMD00621066 | SAMD00621203 | SAMD00621340 |
|  | SAMD00620937 | SAMD00621067 | SAMD00621204 | SAMD00621341 |
|  | SAMD00620938 | SAMD00621068 | SAMD00621205 | SAMD00621342 |
|  | SAMD00620941 | SAMD00621069 | SAMD00621206 | SAMD00621344 |
|  | SAMD00620944 | SAMD00621070 | SAMD00621208 | SAMD00621345 |
|  | SAMD00620946 | SAMD00621071 | SAMD00621209 | SAMD00621346 |
|  | SAMD00620947 | SAMD00621072 | SAMD00621211 | SAMD00621347 |
|  | SAMD00620951 | SAMD00621073 | SAMD00621212 |  |
|  | SAMD00620952 | SAMD00621074 | SAMD00621215 |  |
|  | SAMD00620953 | SAMD00621075 | SAMD00621216 |  |
|  | SAMD00620954 | SAMD00621078 | SAMD00621217 |  |
|  | SAMD00620955 | SAMD00621079 | SAMD00621218 |  |
|  | SAMD00620957 | SAMD00621080 | SAMD00621219 |  |
|  | SAMD00620959 | SAMD00621081 | SAMD00621220 |  |
|  | SAMD00620960 | SAMD00621082 | SAMD00621221 |  |
|  | SAMD00620961 | SAMD00621083 | SAMD00621223 |  |
|  | SAMD00620962 | SAMD00621084 | SAMD00621224 |  |
|  | SAMD00620963 | SAMD00621086 | SAMD00621225 |  |
|  | SAMD00620965 | SAMD00621087 | SAMD00621226 |  |
|  | SAMD00620966 | SAMD00621088 | SAMD00621227 |  |
|  | SAMD00620967 | SAMD00621089 | SAMD00621228 |  |
|  | SAMD00620968 | SAMD00621090 | SAMD00621229 |  |
|  | SAMD00620970 | SAMD00621091 | SAMD00621230 |  |
|  | SAMD00620971 | SAMD00621093 | SAMD00621232 |  |
|  | SAMD00620972 | SAMD00621094 | SAMD00621233 |  |
|  | SAMD00620973 | SAMD00621095 | SAMD00621234 |  |
|  | SAMD00620974 | SAMD00621096 | SAMD00621235 |  |
|  | SAMD00620975 | SAMD00621097 | SAMD00621236 |  |
|  | SAMD00620976 | SAMD00621098 | SAMD00621241 |  |
|  | SAMD00620979 | SAMD00621099 | SAMD00621242 |  |
|  | SAMD00620980 | SAMD00621102 | SAMD00621246 |  |
|  | SAMD00620981 | SAMD00621103 | SAMD00621247 |  |
|  | SAMD00620982 | SAMD00621105 | SAMD00621248 |  |
|  | SAMD00620983 | SAMD00621106 | SAMD00621250 |  |
|  | SAMD00620984 | SAMD00621108 | SAMD00621251 |  |
|  | SAMD00620985 | SAMD00621110 | SAMD00621252 |  |
|  | SAMD00620986 | SAMD00621111 | SAMD00621254 |  |
|  | SAMD00620987 | SAMD00621113 | SAMD00621255 |  |
|  | SAMD00620989 | SAMD00621116 | SAMD00621256 |  |
