# Supplementary figures and images for: Prediction and causal inference of hyperuricemia using gut microbiota
Source: Sci Rep. 2024 Apr 30;14:9901. doi: 10.1038/s41598-024-60427-6 (PMC11061287; doi:10.1038/s41598-024-60427-6)

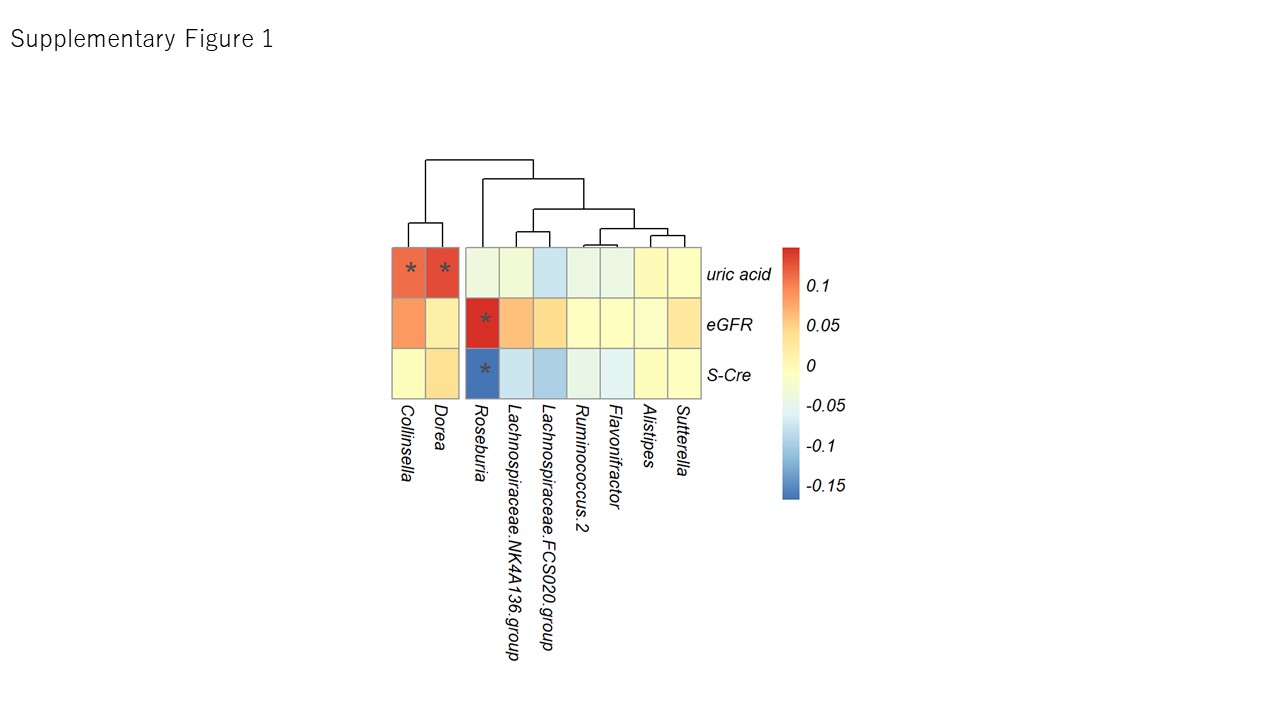

Supplement: Supplementary file 3 — Supplementary Figure S1. [file 41598_2024_60427_MOESM3_ESM.jpg]

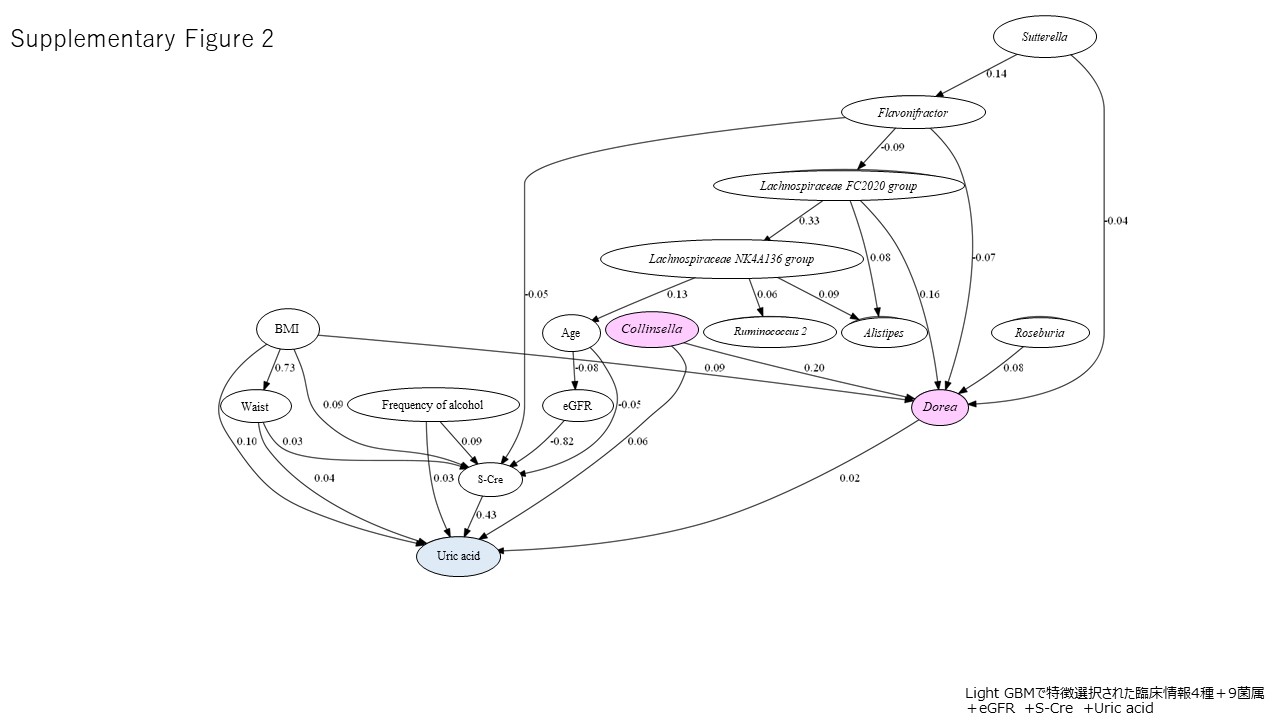

Supplement: Supplementary file 4 — Supplementary Figure S2. [file 41598_2024_60427_MOESM4_ESM.jpg]
